# Evaluation of the Diagnostic Utility of the Traditional and Revised WHO Dengue Case Definitions

**DOI:** 10.1371/journal.pntd.0002385

**Published:** 2013-08-22

**Authors:** Gamaliel Gutiérrez, Lionel Gresh, María Ángeles Pérez, Douglas Elizondo, William Avilés, Guillermina Kuan, Ángel Balmaseda, Eva Harris

**Affiliations:** 1 Sustainable Sciences Institute, Managua, Nicaragua; 2 Hospital Infantil Manuel de Jesús Rivera, Ministry of Health, Managua, Nicaragua; 3 Centro de Salud Sócrates Flores Vivas, Ministry of Health, Managua, Nicaragua; 4 Laboratorio Nacional de Virología, Centro Nacional de Diagnóstico y Referencia, Ministry of Health, Managua, Nicaragua; 5 Division of Infectious Diseases and Vaccinology, School of Public Health, University of California, Berkeley, Berkeley, California, United States of America; RTI International, United States of America

## Abstract

Dengue, a mosquito-borne viral illness, is a major public health problem worldwide, and its incidence continues to increase. In 2009, the World Health Organization published guidelines that included a revision of the dengue case definition. Compared to the traditional definition, the revised case definition relies more on signs than on symptoms, making it more applicable to young children. We evaluated the diagnostic utility of both case definitions in two studies of pediatric dengue in Managua, Nicaragua. In a community-based cohort study, we included data from 3,407 suspected dengue cases, of which 476 were laboratory-confirmed. In the second study, we collected information from 1,160 participants recruited at the national pediatric reference hospital (723 laboratory-confirmed). In the cohort study, the traditional definition had 89.3% sensitivity and 43.1% specificity, while the revised definition yielded similar sensitivity (86.6%) and higher specificity (55.2%, p<0.001). In the hospital study, the traditional case definition yielded 96.7% sensitivity and 22.0% specificity, whereas the revised case definition had higher sensitivity (99.3%, p<0.001) but lower specificity (8.5%, p<0.001). We then evaluated the performance of two diagnostic models based on the signs/symptoms included in each definition by analyzing the effect of increasing numbers of signs/symptoms on the sensitivity and specificity of case capture. Receiver operating characteristic analysis showed a slightly better performance for the revised model in both studies. Interestingly, despite containing less symptoms that cannot be readily expressed by children aged less than 4 years, the revised definition did not perform better in this age group. Overall, our results indicate that both case definitions have similar capacity to diagnose dengue. Owing to their high sensitivity and low specificity, they should be primarily used for screening purposes. However, in a primary care setting, neither of the case definitions performed well as a screening test in younger children.

## Author Summary

Dengue, a mosquito-borne viral infectious disease, is a major health problem worldwide. Its incidence has dramatically increased in the last 3 decades, particularly in the Americas. The traditional World Health Organization dengue case definition was in use for over 30 years, but in 2009 a revised dengue case definition, containing fewer symptoms and more signs, was published. In this study, we evaluate the diagnostic utility of both case definitions in children in Managua, Nicaragua. We used clinical data from suspected dengue cases enrolled at two different sites: a health center and the national pediatric reference hospital. Laboratory tests were performed to determine dengue virus infection status. At both sites, the case definitions showed high sensitivity (≥85%) and low specificity (8–55%). In children under 4 years old, who cannot express some of the symptoms included in the definitions, both case definitions showed reduced sensitivity and increased specificity. This reduction was particularly important in the health center, where sensitivity dropped below 50%. In conclusion, both case definitions should be primarily used for screening and not for diagnosis, and caution must be exercised when applying the definitions to younger children. This is the first published evaluation of the revised dengue case definition in pediatric populations.

## Introduction

Dengue is caused by the four serotypes of dengue virus (DENV-1-4), a flavivirus transmitted primarily by *Aedes aegypti* and *Ae. albopictus* mosquitoes. Dengue is recognized as a major health problem globally, causing an estimated 100 million cases in over 100 countries in Asia, Africa and the Americas [1]–[3]. Even though dengue disease burden is highest in Asia, the Americas have experienced a dramatic increase in the number of reported cases over the last thirty years [4], [5].

Fever is a common symptom among children seeking medical care. Differentiating dengue from other febrile illnesses is key to providing timely and appropriate care [6]. In response to outbreaks of dengue fever in the 1950s and 1960s in many countries of the Asia-Pacific region, the World Health Organization (WHO) developed guidelines for dengue diagnosis, treatment and control which were approved in 1975 [7], [8]. These guidelines defined a probable dengue case as an acute febrile illness accompanied by two or more of the following criteria: headache, retro-orbital pain, myalgia, arthralgia, rash, hemorrhagic manifestations and leukopenia. The first four criteria are symptoms and thus, by definition, are reported by the patient and cannot be measured directly by the physician. Moreover, these symptoms cannot be easily verbalized by younger children, particularly those under 4 years [9].

In 2009, the WHO published new guidelines for dengue diagnosis, treatment, prevention and control [6], which includes a revised dengue case definition. In these guidelines, a probable dengue case was defined as fever and two of the following criteria: nausea/vomiting, rash, aches and pains, positive tourniquet test, leukopenia, and any warning sign (abdominal pain or tenderness, persistent vomiting, clinical fluid accumulation, mucosal bleeding, lethargy/restlessness, liver enlargement >2 cm, or increase in hematocrit concurrent with rapid decrease in platelet count) [6]. In this revised case definition, only one symptom – aches and pains – is included, the remaining criteria are signs that are evaluated by a healthcare professional.

Several studies have assessed the performance of the traditional dengue case definition in Thailand, Singapore, Peru, and Colombia [9]–[11]. Overall, the definition had high sensitivity (85–100%) and low specificity (2–36%). As such, it was determined to be a good screening method. However, its low specificity leads to over-diagnosis if not accompanied by laboratory confirmation, which is not always available in resource-limited settings [9]. On the contrary, only one evaluation of the revised case definition has been reported; namely, a study performed in an adult population that yielded a sensitivity of 80% and a specificity of 57% [12].

Here, we compared the diagnostic utility of the traditional and revised WHO dengue case definitions in two different settings. First, we analyzed data from suspected dengue cases identified in a prospective pediatric cohort study based at primary health care center in Managua, Nicaragua, from 2004 to 2011 [13], [14]. Second, we examined participants enrolled in a prospective study of dengue based at the National Pediatric Reference Hospital in Managua, Nicaragua, from 2005 to 2012 [15]–[19]. In each study, we evaluated the sensitivity, specificity and predictive values of the individual signs and symptoms and of the traditional and revised dengue case definitions. In addition, we carried out receiver operating characteristic (ROC) analyses and tested the performance of both case definitions in younger versus older children.

## Materials and Methods

### Ethics statement

The protocols for the Pediatric Dengue Cohort Study and the hospital-based study were reviewed and approved by the Institutional Review Boards (IRB) of the University of California, Berkeley, and of the Nicaraguan Ministry of Health. Parents or legal guardians of all subjects in both studies provided written informed consent, and subjects 6 years of age and older provided assent. In the hospital study, children 6 years of age and older displaying signs of altered consciousness at the time of recruitment and thus unable to provide assent were excluded.

### Cohort study

The Pediatric Dengue Cohort Study is an ongoing community-based prospective study established in 2004 in Managua, Nicaragua [13], [14]. The study is based at the local municipal health center, the Health Center Sócrates Flores Vivas (HCSFV). The area served by the HCSFV is District II of Managua, a low- to middle-income area with a population of approximately 62,500. In August–September 2004, children aged two to nine years old living in District II were enrolled, and new participants have been enrolled each year since then to maintain the cohort age structure. Children are withdrawn from the study when they reach 15 years of age. Participants are encouraged to present at the first sign of illness to the HCSFV, where study physicians provide medical care and screen for signs and symptoms of dengue. Suspected dengue cases, as defined by the traditional WHO dengue case definition [8] and febrile participants without other apparent origin (undifferentiated febrile illnesses) who present in the first six days of illness are screened by serological, molecular and virological methods for acute DENV infection [13], [14]. A convalescent-phase blood sample (two weeks after onset of fever) is also collected for serological assays. Participants are followed during the acute phase of illness by study physicians at the HCSFV. Clinical data, including signs, symptoms, and treatment, are recorded at every visit. Data collected from August 2004 to December 2011 was used for this analysis. The presence or absence of each criterion for both case definitions was evaluated throughout the course of disease.

### Hospital-based study

An ongoing hospital-based prospective study of dengue to study clinical, immunological and viral risk factors for severe dengue was established in 1998 in the Infectious Disease Ward of the Hospital Infantil Manuel de Jesús Rivera Hospital (HIMJR), the national pediatric reference hospital in Managua [15]–[20]. In-patients and out-patients between 6 months and 14 years of age are enrolled when they present at the HIMJR with documented or reported fever of less than 7 days and one or more of the following signs and symptoms: headache, arthralgia, myalgia, retro-orbital pain, positive tourniquet test, petechiae or others signs of bleeding. Enrollment occurs each year during the peak of the dengue season (August–January). Children with a defined focus other than dengue are excluded, as well as children weighing less than 8 kg. Upon enrollment, a medical history is taken, and a complete physical exam is performed. Acute blood samples are collected daily for complete blood count and serological, virological, and molecular testing for DENV infection. A convalescent-phase blood sample (two weeks after onset of fever) is also collected. Both in-patient and out-patient subjects are followed clinically through the acute phase of illness. Clinical data, including signs, symptoms, and treatment, are recorded daily through ambulatory follow-up visits for out-patients, and at least every 12 hours for in-patients during hospitalization. All data is collected on standardized forms. Participants are followed for 3 to 8 days. Participants requiring more intensive therapies are transferred to the intensive care unit. Data collected from August 2005 to January 2012 was used for analysis. The presence or absence of each criterion for both case definitions was evaluated over the course of disease evolution.

### Laboratory-confirmed dengue cases

For both studies, a participant was considered positive for DENV infection when laboratory tests met one or more of the following criteria: 1) dengue viral RNA was detected by RT-PCR [21], [22]; 2) DENV was isolated [21]; 3) seroconversion of DENV-specific IgM was detected by MAC-ELISA in paired acute and convalescent samples [21], [23]; and 4) DENV-specific antibody titer by Inhibition ELISA [20], [24], [25] demonstrated a 4-fold or greater increase between acute and convalescent sera. Primary DENV infections were considered those in which the convalescent antibody titer was <2,560, and secondary infections were considered those in which the convalescent antibody titer was ≥2,560 as determined by Inhibition ELISA. A case was considered indeterminate if RT-PCR yielded negative results, no DENV was isolated and a convalescent sample could not be obtained. Indeterminate cases were excluded from this analysis.

### Dengue signs, symptoms, and case definitions

Signs and symptoms were defined and measured as described in Table 1. As children under 4 years old cannot easily express aches, reporting of headache, retro-orbital pain, myalgia, arthralgia, and aches and pains was only considered for children aged 4 years old or more. Dengue cases were defined according to WHO criteria. The traditional dengue case definition was the presence of fever (or history of fever) plus two or more of the following: headache, retro-orbital pain, myalgia, arthralgia, rash, hemorrhagic manifestations and leukopenia (white blood cell count <5,000 cells/mm^3^) [7], [8]. The revised dengue case definition was the presence of fever (or history of fever) plus two or more of the following: nausea/vomiting, rash, aches and pains, leukopenia, positive tourniquet test (petechia ≥20 per inch^2^) and any warning sign (abdominal pain or tenderness, persistent vomiting, clinical fluid accumulation, mucosal bleeding, lethargy/restlessness, liver enlargement >2 cm, or increase in hematocrit concurrent with rapid decrease in platelet count) [6] (Table 1).

**Table 1:** Traditional and revised WHO dengue case and criteria definitions.

Terms	Definition
Suspected dengue case (Traditional definition)	Fever for less than 7 days plus two or more of the following criteria: headache, retro-orbital pain, myalgia, arthralgia, rash, hemorrhagic manifestations, leukopenia
Suspected dengue case (Revised definition)	Fever for less than 7 days plus two or more of the following criteria: nausea/vomiting, rash, aches and pains, positive tourniquet test, leukopenia, any warning sign
Fever	Temperature ≥37.8°C as recorded by study personnel or history of fever
Headache, retro-orbital pain, myalgia and arthralgia	Symptoms reported by participants aged ≥4 years old
Rash	Change of color, appearance or texture of the skin observed by a study physician
Hemorrhagic manifestations	Spontaneous petechiae, purpura, ecchymosis, hematoma, hemoptysis, epistaxis, gingival bleeding, melena, hematemesis, hematuria, subconjunctival hemorrhage, menorrhagia, or vaginal bleeding as observed by a study physician or reported by the patient, or positive tourniquet test
Leukopenia	White blood count ≤5,000 cells/mm^3^
Nausea/vomiting	Nausea or at least one emesis reported by the patient or observed by a study physician
Aches and pains	Any of the following: headache, retro-orbital pain, myalgia or arthralgia
Positive tourniquet test	≥20 petechiae/inch^2^
Warning signs	Abdominal pain or tenderness, persistent vomiting, clinical fluid accumulation, mucosal bleeding, lethargy/restlessness, liver enlargement, increase in hematocrit concurrent with rapid decrease in platelet count
Abdominal pain or tenderness	Pain in the abdominal region reported spontaneously by the patient or when palpated by a study physician
Persistent vomiting	Three or more emesis in a period of one hour, or five or more in a period of six hours
Clinical fluid accumulation	Peri-orbital, facial or lower limb edema as reported by the study physician, or pleural effusion, ascites or gall-bladder wall thickening (≥3 mm) as observed via X-ray radiography or ultrasonography
Mucosal bleeding	Any of the following: hemoptysis, epistaxis, gingival bleeding, melena, hematemesis, hematuria, menorrhagia, vaginal bleeding, or subconjunctival hemorrhage as observed by a study physician or reported by the patient
Lethargy/restlessness	Glasgow coma scale score <15 for children aged 5 years or more or Blantyre coma scale <5 for children under 5, as evaluated by a study physician
Liver enlargement	Liver enlarged >2 cm below the edge of the ribs as palpated by a study physician
Increase in hematocrit concurrent with rapid decrease in platelet count	Decrease in platelet count >10,000 platelets/mm^3^ in 24 hours, or increase in hematocrit accompanied by a platelet count <100,000 platelets/mm^3^

### Statistical analysis

For both studies, signs and symptoms presented at any time over the course of the disease were included in the analysis. A Chi-squared test was used to associate categorical variables with dengue laboratory results. To determinate sensitivity, specificity and predictive values of each criterion for dengue diagnosis, the laboratory result was considered as the gold standard. A criterion with a sensitivity and specificity of 80% or more was considered of high diagnostic value [26]. ROC (Receiver operator characteristic) analysis was carried by giving one point per criterion present (maximum of 7 points/criteria for the traditional case definition and maximum of 6 points/criteria for the revised case definition). For each point, the sensitivity, specificity and accuracy for diagnosing dengue was calculated. The value of the area under the curve (AUC) was also computed. A test with an AUC value between 0.51 and 0.70 was considered of poor diagnostic value; from 0.71 to 0.90 as useful for some purposes; and >0.90 as of high diagnostic value [27]. All data analyses were performed using Intercooled Stata 9.0 (StataCorp LP, College Station, Texas), with a 95% confidence level.

## Results

### Study population

Cases were identified in the Pediatric Dengue Cohort Study and a hospital-based study, both based in Managua, Nicaragua. In the cohort study, 3,617 cases were identified from August 2004 to December 2011 (Table 2). A total of 3,407 cases were included in the analysis. Two hundred and ten cases did not have a confirmed positive or negative laboratory result and were excluded from the analysis (see Methods). The median age of cases was 7.0 years (interquartile range (IQR): 5.0–9.0 years). Cases were equally distributed among females (48.9%) and males (51.1%) (Z-test p = 0.145). The median day of illness at presentation was 2 (IQR: 1–2). A total of 476 (14.0%) cases were laboratory-confirmed DENV infections. Among laboratory-confirmed cases, 50.4% experienced a secondary DENV infection. Infecting DENV serotypes were detected by RT-PCR and confirmed by virus isolation. Most infections were caused by DENV-3 (52.3%) followed by DENV-2 (27.7%) and DENV-1 (12.0%). In 35 (7.4%) of the confirmed dengue cases, the infecting serotype was not identified. In the hospital study, 1,210 participants were enrolled from August 2005 to January 2012 (Table 2). Fifty (4.1%) participants had indeterminate results in the diagnostic assays and were excluded from the analysis. Information from the remaining 1,160 participants was used for analysis. The median participant age was 7.9 years (IQR: 4.9–11.1 years). Participants were equally distributed among females (47.8%) and males (52.2%) (Z-test p = 0.142). The median day of illness at enrollment was 4 (IQR: 3–5). A total of 723 (62.3%) cases were confirmed DENV infections. Of these, 53.1% were secondary infections. The infecting DENV serotype was mainly DENV-3 (58.9%) followed by DENV-2 (22.8%) and to a lesser extent DENV-1 (7.5%) (Table 2). The infecting DENV serotype was not identified in 77 (10.7%) of the confirmed dengue cases.

**Table 2:** General characteristics of the study population.

Characteristics	Cohort study (# %)	Hospital study (# %)
Cases evaluated	3,617	1,210
Cases included in the analysis	3,407	1,160
Laboratory-confirmed dengue	476 (14.0)	723 (62.3)
Age in years – median (interquartile range, IQR)	7.0 (5.0–9.0)	7.9 (4.9–11.1)
<1 year	—	50 (4.3)
1–3 years	481 (14.1)	198 (17.1)
4–9 years	2,100 (61.7)	521 (44.9)
10–14 years	826 (24.2)	391 (33.7)
Sex		
Female	1,661 (48.9)	555 (47.8)
Day of illness at presentation (median, IQR)Immune response^a^	2 (1–2)	4 (3–5)
Primary	222 (46.6)	306 (42.3)
Secondary	240 (50.4)	384 (53.1)
Indeterminate	14 (3.0)	33 (4.6)
Dengue virus serotype^a^		
DENV-1	57 (12.0)	54 (7.5)
DENV-2	132 (27.7)	165 (22.8)
DENV-3	249 (52.3)	426 (58.9)
DENV-4	1 (0.2)	—
DENV-1 & DENV-2 co-infection	1 (0.2)	—
DENV-1 & DENV-4 co-infection	1 (0.2)	—
DENV-3 & DENV-4 co-infection	—	1 (0.1)
Unknown	35 (7.4)	77 (10.7)

a Among laboratory-confirmed dengue cases.

### Diagnostic value of traditional and revised dengue case definition signs and symptoms

Using clinical data collected in both the cohort and the hospital-based studies, we compared the diagnostic utility of the revised versus the traditional WHO case definitions. First, we analyzed the association of each sign and symptom of both dengue case definitions (Table 1) with the positivity of dengue diagnosis as defined by laboratory testing, and calculated their sensitivity, specificity, positive predictive value (PPV) and negative predictive value (NPV).

In the cohort study, all signs and symptoms (criteria) were significantly associated with laboratory-confirmed dengue (chi-square test p<0.05) (Table 3). However, no single criterion showed both a high sensitivity and a high specificity. For instance, aches and pains and headache were very frequent in patients with dengue (sensitivity >85%), but also in patients without dengue (specificity <25%) (Table 3). On the contrary, nausea and vomiting, positive tourniquet test and rash, had high specificity but low sensitivity. The remaining signs and symptoms had sensitivities ranging from 40 to 70% and specificities ranging from 60 to 80%. Strikingly, all criteria had an NPV over 85%, meaning that the absence of these signs/symptoms could be used to rule out dengue. However, the PPV was consistently low (15–35%; except for rash, 55%).

**Table 3:** Diagnostic values of the traditional and revised dengue case definition signs and symptoms, cohort study.

Signs/symptoms	Sensitivity % (95% CI)	Specificity % (95% CI)	PPV % (95% CI)	NPV % (95% CI)
Aches and pains^a^^,^ b	91.8 (88.9–94.2)	18.4 (16.9–20.0)	16.7 (15.2–18.2)	92.7 (90.1–94.8)
Headache^a^^,^ b	86.8 (83.3–89.9)	22.2 (20.6–23.9)	16.5 (15.0–18.1)	90.5 (87.9–92.7)
Leukopenia^b^	68.3 (63.9–72.4)	79.9 (78.4–81.3)	35.5 (32.4–38.7)	93.9 (92.9–94.8)
Any warning sign^b^	66.4 (61.9–70.6)	63.6 (61.9–65.4)	22.9 (20.7–25.2)	92.1 (90.8–93.2)
Arthralgia^a^^,^ b	56.5 (51.7–61.1)	61.1 (59.1–63.0)	20.5 (18.2–22.9)	88.8 (87.2–90.2)
Myalgia^a^^,^ b	52.4 (47.6–57.1)	66.4 (64.5–68.3)	21.7 (19.2–24.3)	88.7 (87.2–90.1)
Hemorrhagic manifestations^b^	52.1 (47.5–56.7)	76.7 (75.2–78.3)	26.7 (23.8–29.6)	90.8 (89.6–91.9)
Retro-orbital pain^a^^,^ b	41.7 (37.1–46.5)	73.8 (72.0–75.5)	22.0 (19.2–25.0)	87.7 (86.2–89.1)
Positive tourniquet test^b^^,^ c	39.9 (35.5–44.5)	88.2 (87.0–89.4)	35.6 (31.6–39.9)	90.0 (88.8–91.0)
Rash^b^	29.6 (25.6–33.9)	96.1 (95.3–96.8)	55.3 (49.0–61.5)	89.4 (88.2–90.4)
Nausea/vomiting^b^	27.1 (23.2–31.3)	82.2 (80.7–83.5)	19.8 (16.8–23.1)	87.4 (86.1–88.6)

a Children under 4 years were excluded from the analysis. N for negative cases = 2,485; N for positive cases = 441.

b Signs and symptoms associated with dengue (p<0.05).

c 23 with missing data among negative cases (N = 2,908).

In the hospital-based study, only six of the 11 criteria were associated with dengue, namely any warning sign, rash, leukopenia, hemorrhagic manifestations, positive tourniquet test and nausea/vomiting (chi-square test p<0.05) (Table 4). However, none of these criteria had both high sensitivity and high specificity. Any warning sign, rash, leukopenia and hemorrhagic manifestations showed a high sensitivity (>80%) but a low specificity (15–53%) (Table 4). Positive tourniquet test was the only criterion with high specificity (80.3%) but its sensitivity was low (49.7%), while nausea/vomiting had low sensitivity (48.7%) and specificity (57.9%). The remaining criteria (aches and pains, headache, retro-orbital pain, myalgia and arthalgia) were not associated with laboratory diagnosis of dengue. None of the signs and symptoms yielded high predictive values, except for positive tourniquet test, which had a PPV of 80.7% (Table 4).

**Table 4:** Diagnostic values of the traditional and revised dengue case definition signs and symptoms, hospital-based study.

Signs/symptoms	Sensitivity % (95% CI)	Specificity % (95% CI)	PPV % (95% CI)	NPV % (95% CI)
Any warning sign^b^	92.8 (90.7–94.6)	15.6 (12.3–19.3)	64.5 (61.5–67.4)	56.7 (47.3–65.7)
Rash^b^	91.7 (89.4–93.6)	36.2 (31.6–40.9)	70.4 (67.4–73.3)	72.5 (66.0–78.3)
Leukopenia^b^	83.1 (80.2–85.8)	49.4 (44.6–54.2)	73.1 (69.9–76.1)	63.9 (58.5–69.0)
Aches and pains^a^	82.0 (78.7–84.9)	20.8 (16.4–25.7)	67.0 (63.5–70.4)	37.0 (29.8–44.7)
Hemorrhagic manifestations^b^	80.6 (77.6–83.5)	52.6 (47.8–57.4)	73.8 (70.6–76.8)	62.2 (57.0–67.1)
Headache^a^	74.5 (70.8–77.9)	26.6 (21.8–31.9)	66.6 (62.9–70.1)	34.7 (28.7–41.2)
Positive tourniquet test^b^	49.7 (45.9–53.4)	80.3 (76.3–83.9)	80.7 (76.7–84.2)	49.1 (45.4–52.8)
Nausea/vomiting^b^	48.1 (44.4–51.8)	57.9 (53.1–62.6)	65.4 (61.2–69.5)	40.3 (36.4–44.2)
Retro-orbital pain^a^	41.6 (37.6–45.6)	61.0 (55.3–66.5)	67.7 (62.6–72.4)	34.8 (30.7–38.9)
Myalgia^a^	36.1 (32.3–40.1)	69.2 (63.7–74.3)	69.6 (64.2–74.7)	35.6 (31.7–39.5)
Arthralgia^a^	29.5 (25.9–33.3)	72.7 (67.4–77.6)	67.9 (61.9–73.5)	34.5 (30.8–38.3)

a Children under 4 years were excluded from the analysis. N for negative cases = 308; N for positive cases = 604.

b Signs and symptoms associated with dengue (p<0.05).

### Receiver Operating Characteristics analysis

Next, we carried out a receiver operating characteristics (ROC) analysis of two diagnostic models based on the criteria included in the traditional and revised dengue case definitions. For each definition, we calculated the sensitivity, specificity and accuracy of the presence of only fever, fever plus one or more criteria, fever plus two or more criteria, etc. (Tables 5 and 6). By definition, the sensitivity, specificity and accuracy of the WHO dengue case definitions *per se* correspond to those calculated for fever plus two or more criteria.

**Table 5:** Receiver operating characteristics (ROC) analysis of two diagnostics models based on the list of criteria included in each dengue case definition, cohort study.

Number of criteria	Traditional dengue case definition			Revised dengue case definition		
	Sensitivity	Specificity	Accuracy	Sensitivity	Specificity	Accuracy
Fever only	100.0	0.0	14.0	100.0	0.0	14.0
Fever plus 1 or more criteria	97.1	15.7	27.0	96.0	15.6	26.9
**Fever plus 2 or more criteria** a	89.3	43.1	49.6	86.6	55.2	59.6
Fever plus 3 or more criteria	76.7	61.2	63.4	65.6	83.3	80.8
Fever plus 4 or more criteria	59.5	76.8	74.4	36.6	96.8	88.4
Fever plus 5 or more criteria	36.8	91.9	84.2	11.0	99.8	87.5
Fever plus 6 or more criteria	15.6	98.5	86.9	2.3	100.0	86.3
Fever plus 7 criteria	4.8	99.7	86.4	—	—	—

a Dengue case definition.

**Table 6:** Receiver operating characteristics (ROC) analysis of two diagnostics models based on the list of criteria included in each dengue case definition, hospital-based study.

Number of criteria	Traditional dengue case definition			Revised dengue case definition		
	Sensitivity	Specificity	Accuracy	Sensitivity	Specificity	Accuracy
Fever only	100.0	0.0	62.3	100.0	0.0	62.3
Fever plus 1 or more criteria	99.6	5.3	64.1	99.9	2.5	63.2
**Fever plus 2 or more criteria** a	96.7	22.0	68.5	99.3	8.5	65.1
Fever plus 3 or more criteria	85.9	48.5	71.8	93.8	29.1	69.4
Fever plus 4 or more criteria	62.0	66.1	63.5	78.7	60.0	71.6
Fever plus 5 or more criteria	35.7	82.4	53.5	48.0	85.4	62.1
Fever plus 6 or more criteria	20.9	91.8	47.6	16.6	95.9	46.5
Fever plus 7 criteria	11.8	97.5	44.1	—	—	—

a Dengue case definition.

**Figure 1:**
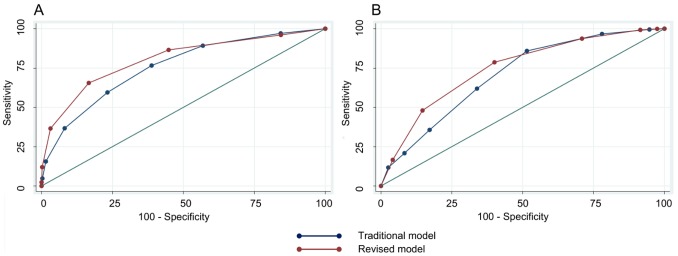
Receiver operating characteristic (ROC) curves of two diagnostic models based on the criteria included in each dengue case definition. (A) Cohort study. The area under the curve (AUC) of the ROC curve was slightly higher for the revised model (0.80) than for the traditional model (0.75) (chi-square test p<0.001). (B) Hospital-based study. The AUC was slightly higher for the revised model (0.75) than for the traditional model (0.71) (chi-square test p<0.001).

As expected, the sequential addition of criteria decreased sensitivity and increased specificity (Tables 5 and 6). In the cohort study, the ROC analysis based on the traditional dengue case definition showed that the highest percentage of cases correctly classified (accuracy) was for fever plus 6 or more criteria (86.9%). For this number of signs/symptoms, the specificity was high (98.5%), but the corresponding sensitivity very low (15.6%) (Table 5). For the revised dengue case model, the highest accuracy was observed for fever and 4 or more criteria (88.4%), with a high specificity (96.8%) but again a low sensitivity (36.6%). No data point in the ROC analysis showed both a high sensitivity (>80%) and high specificity (>80%). The area under the curve (AUC) of the ROC curve was slightly higher (chi-square test p<0.001) for the revised model, 0.80 (95%CI 0.78–0.82), than for the traditional model, 0.75 (95%CI 0.73–0.78) (Fig. 1A).

In the hospital study, the greatest accuracy was measured for fever plus 3 or more criteria in the traditional model (71.8%) with a sensitivity of 85.9% and a specificity of 48.5% (Table 6). For the revised model, the greatest accuracy was observed for fever plus 4 or more criteria (71.6%) with a sensitivity of 78.7% and a specificity of 60.0%. As in the cohort study, no data point in the ROC analysis showed both a high sensitivity and high specificity. The area under the ROC curve (AUC) was 0.71 (95%CI 0.67–0.73) for the traditional model, and slightly higher (chi-square test p<0.001) for the revised model, 0.75 (95%CI 0.71–0.77) (Fig. 1B).

### Effect of age in the diagnostic utility of the dengue case definitions

We then evaluated the diagnostic utility of the traditional and revised dengue case definitions. Sensitivity and specificity as well as positive and negative predictive values were calculated for each case definition. In the cohort study, the traditional case definition had a sensitivity of 89.3% and a specificity of 43.1% (Table 7). The NPV was high (96.1%) and the PPV low (20.3%). We then stratified this analysis by age, taking into account that symptoms such as arthralgia, headache, myalgia, and retro-orbital pain can only be reliably reported by children aged 4 years or more. In children under the age of 4, for whom these symptoms were not included, the sensitivity dropped to 37.1%, 2.5-fold lower that in children over 4 (91.4%) (Table 7). On the contrary, the specificity was higher in the younger age group compared to the older (95.3% versus 39.4%). Comparable observations were made for the revised case definition. Overall, the revised definition had similar sensitivity, PPV and NPV when compared to the traditional definition. Its specificity was higher to some extent (55.2% versus 43.1%, McNemar test p<0.001). When stratifying by age, children under the age of 4 showed a strong reduction in sensitivity and an increase in specificity (Table 7). This effect was observed despite the fact that in the revised dengue case definition, only one criterion is not accurately reported by children under 4 years old (aches and pains).

**Table 7:** Diagnostic values of the traditional and revised WHO dengue case definitions by age, cohort study.

WHO Dengue case definition	Sensitivity % (95% CI)	Specificity % (95% CI)	PPV % (95% CI)	NPV % (95% CI)
**Traditional case definition**				
All patients	89.3 (86.2–91.9)	43.1 (41.3–44.9)	20.3 (18.6–22.1)	96.1 (94.9–97.1)
Children <4 years old	37.1 (21.5–55.1)	95.3 (92.9–97.1)	38.2 (22.2–56.4)	95.1 (92.1–96.9)
Children ≥4 years old	91.4 (88.4–93.8)	39.4 (37.5–41.3)	21.1 (19.3–23.0)	96.3 (94.9–97.3)
**Revised case definition**				
All patients	86.6 (83.2–89.5)	55.2 (53.4–57.0)	23.9 (21.9–26.0)	96.2 (95.2–97.1)
Children <4 years old	45.7 (28.8–63.4)	80.5 (76.5–84.1)	15.7 (9.2–24.2)	94.9 (92.2–96.9)
Children ≥4 years old	91.2 (88.1–93.6)	45.9 (44.5–48.5)	23.3 (21.4–25.4)	96.7 (95.5–97.7)

In the hospital study, the traditional definition showed a very high sensitivity (96.7%) and a low specificity (22.0%) (Table 8). Its positive and negative predictive values were 67.2% and 80.0%, respectively. In children under 4 years old, the sensitivity decreased slightly while the specificity improved. The revised case definition had an even higher sensitivity (99.3%, McNemar test p<0.001) and a lower specificity (8.5%, McNemar test p<0.001) than the traditional definition (Table 8). Similar to the traditional definition, the sensitivity in children under the age of 4 showed a marginal decrease and the specificity was increased when compared to older children.

**Table 8:** Diagnostic values of the traditional and revised WHO dengue case definitions by age, hospital-based study.

WHO Dengue case definition	Sensitivity % (95% CI)	Specificity % (95% CI)	PPV % (95% CI)	NPV % (95% CI)
**Traditional case definition**				
All patients	96.7 (95.1–97.9)	22.0 (18.2–26.1)	67.2 (64.3–70.1)	80.0 (71.7–86.7)
Children <4 years old	87.4 (80.1–92.8)	50.4 (41.5–59.3)	61.9 (54.1–69.3)	81.3 (71.0–89.1)
Children ≥4 years old	98.5 (97.2–99.3)	12.7 (9.2–16.9)	68.9 (65.7–71.9)	81.3 (67.4–91.1)
**Revised case definition**				
All patients	99.3 (99.2–100.0)	8.5 (6.0–11.5)	64.2 (61.3–67.0)	88.1 (74.4–96.0)
Children <4 years old	96.6 (91.6–99.1)	22.5 (15.6–30.7)	53.5 (46.6–60.3)	87.9 (71.8–96.6)
Children ≥4 years old	99.8 (99.1–100.0)	3.9 (2.0–6.7)	67.1 (63.9–70.1)	92.3 (64.0–99.8)

## Discussion

Here, we compared the traditional WHO dengue case definition, which was implemented for over 40 years, and the revised definition approved in 2009. The case definitions were compared in two distinct pediatric studies in Managua, Nicaragua: a prospective cohort study based at a municipal health care center, and a clinical study based at the national pediatric reference hospital. In the cohort study, the traditional case definition had high sensitivity (89.3%) and low specificity (43.1%), while the revised definition had similar sensitivity (86.6%) and higher specificity (55.2%, p<0.001). In participants under 4 years, both case definitions showed a dramatically reduced sensitivity (37.1% and 45.7% for the traditional and revised definition, respectively) as well as an increased specificity (95.3% and 80.5%, respectively). In the hospital study, the traditional case was also highly sensitive (96.7%) and poorly specific (22.0%), whereas the revised case definition had slightly higher sensitivity (99.3%, p<0.001) but lower specificity (8.5%, p<0.001). In participants under 4 years, both case definitions displayed a reduction in sensitivity (87.4% for the traditional definition and 96.6% for the revised definition) and an increase in specificity (50.4% and 22.5%, respectively).

Several studies have assessed the traditional WHO dengue case definition [9]–[12]. These studies have been conducted in different countries (Thailand [9], Peru [10], Colombia [11], and Singapore [12]) and settings (hospitals [9] and health centers [10]–[12]) and with different inclusion criteria. Three studies relied on clinical data from a single visit, although they differed in the time of disease evolution at the time of inclusion (within 3 [12] or 4 days [10], [11]). Similarly to our study, one study included clinical data from initial and follow-up patient evaluations throughout the course of the disease [9]. Despite these differences, all studies report that the traditional WHO dengue case definition has high sensitivity (85%–100%) and low specificity (2%–36%) to diagnose dengue [9]–[12]. The Singapore study also reported an evaluation of the revised WHO case definition, which yielded a sensitivity of 79.9% and a specificity of 57.0% [12]. However, the study population consisted of adults, and only abdominal pain and mucosal bleeding were included as warning signs.

We also analyzed the utility for dengue diagnosis of each sign and symptom included in the case definitions. In the cohort study, all criteria were significantly associated with dengue (p<0.05). In the hospital study, the only criteria that were not associated with dengue were headache, retro-orbital pain, myalgia, and arthralgia (traditional case definition) and aches and pains (revised case definition). However, despite being statistically associated with confirmed dengue, no single criteria had both high sensitivity and high specificity in either of the studies. Accordingly, none of these criteria are alone, in conjunction with fever, pathognomonic of dengue. The same observation was made by studies in Colombia, Brazil, Peru and Thailand [9]–[11], [28]. We also analyzed whether the inclusion of increasing numbers of criteria in the definition improves the diagnostic utility of the case definitions by creating diagnostic models based on each case definition. The ROC analysis showed that, in both studies, the model based on the revised definition performed slightly better than the traditional. However, a substantial trade-off between sensitivity and specificity was observed as additional criteria were included, and no single number of criteria showed simultaneously high sensitivity and specificity.

The traditional dengue case definition has 7 criteria besides fever [7]. Of these, 4 are symptoms reported by the patient, namely headache, retro-orbital pain, myalgia and arthralgia. Reporting of these symptoms by the patient is more subjective than the evaluation of signs by a health practitioner. Moreover, these symptoms cannot be accurately reported by younger children (generally by those aged less than 4 years), a population at significant risk for dengue [29]. Most studies evaluating the traditional classification have not included this age group [10]–[12]. One study included children aged one to 13 years old but did not stratify its results by age [9]. In a previous report about our cohort study, we found that one-fourth of confirmed pediatric dengue cases did not meet the traditional WHO case definition, and this phenomenon was most pronounced in the youngest children [30]. Consistent with this observation, here we report a reduction in the sensitivity of the traditional case definition when used in children under 4 years old. The reduction was considerable in the cohort study (37.1% versus 91.4% in children aged 4 years and more). Of the 6 criteria included in the revised case definition, only one, aches and pains, is a symptom, which in theory makes this definition more objective and thus more applicable to younger children. However, our analysis showed that the sensitivity of the revised case definition is also dramatically affected by age. In the cohort study, the sensitivity for children aged less than 4 years was twice as low as the sensitivity for older children (45.7% versus 91.2%). Finally, no significant difference in the sensitivity of both case definitions in the younger age group was observed (37.1% and 45.7% for the traditional and revised definition, respectively). Taken together, these results show that despite containing less criteria that cannot be expressed by younger children, the revised case definition does not perform better than the traditional definition in children aged less than 4 years.

Although this analysis was not designed to compare the cohort and hospital studies, the differences in the diagnostic utility of the dengue case definitions are striking. The sensitivity of both case definitions is ∼10 percentage points higher in the hospital study. Their specificity is higher in the cohort study (two- and five-fold higher for the traditional and revised definition, respectively). Several reasons might explain these differences. First and foremost, the inclusion criteria in the studies are different. In the cohort study, suspected dengue [8] and all febrile cases without an apparent cause were studied. In the hospital, febrile cases accompanied by at least one of the following signs or symptoms were included: headache, arthralgia, myalgia, retro-orbital pain, positive tourniquet test, petechiae or others signs of bleeding. Second, the criteria included in the case definitions are measured more frequently in the hospital than at the health center where the cohort study is based. Third, the equipment available to health practitioners in both settings are different. For instance, the use of X-ray radiography or ultrasonography is limited to the hospital. Fourth, more severe cases are seen in the hospital study, whereas the cohort study includes more mild cases. Finally, participants present earlier during the course of illness to the health center than to the hospital.

In summary, we show in two different settings that the revised dengue case definition performs similarly when compared to the traditional case definition. Both case definitions had high sensitivity (over 85%) but low specificity (55% or less). Owing to their diagnostic performance, both case definitions should be primarily used for screening purposes. However, in a primary care setting, neither case definition performed well as a screening test in younger children, as they showed low sensitivity and high specificity. Thus, when using either of the case definitions, particular attention should be paid to younger children. These results further emphasize the need for confirmatory dengue diagnostic methods. At present, laboratory-based testing remains essential both for dengue diagnosis and surveillance. The development of point-of-care tests based on the early detection of DENV antigens (such as the NS1 rapid test [12], [31]) along with the discovery of new biomarkers for dengue could prove invaluable to enhancing dengue diagnosis, clinical management, and surveillance.
